# Intraovarian Platelet-rich Plasma Administration Induced Spontaneous Ovulation in an Anovulatory Woman With PCOS

**DOI:** 10.1210/jcemcr/luad038

**Published:** 2023-04-21

**Authors:** Zaher Merhi, Marco Mouanness, Ruoyu Wang, Serin Seckin

**Affiliations:** Department of Obstetrics and Gynecology, Division of Reproductive Endocrinology and Infertility, SUNY Downstate Health Sciences University, Brooklyn, NY 11203, USA; Rejuvenating Fertility Center, New York, NY 10019, USA; Department of Obstetrics and Gynecology, Maimonides Medical Center, Brooklyn, NY 11219, USA; Rejuvenating Fertility Center, New York, NY 10019, USA; Department of Obstetrics and Gynecology, Division of Reproductive Endocrinology and Infertility, SUNY Downstate Health Sciences University, Brooklyn, NY 11203, USA; Department of Obstetrics and Gynecology, Division of Reproductive Endocrinology and Infertility, Columbia University Medical Center, New York, NY 10019, USA

**Keywords:** PCOS, PRP, anovulation, folliculogenesis, ovaries

## Abstract

The use of platelet-rich plasma (PRP) has become popular as an adjunct to fertility treatment for women with infertility, particularly those with low ovarian reserve and premature ovarian insufficiency. Recent data in a polycystic ovary syndrome (PCOS) animal model demonstrated that intraovarian PRP administration improved folliculogenesis, ovarian antioxidant potential, and serum hormonal imbalance, suggesting that PRP could be considered a novel technique to alleviate PCOS-induced pathogenesis. With injection of PRP into the ovaries, it has been hypothesized that the infusion of cytokines and growth factors may exhibit a local effect that changes the expression of genes important in folliculogenesis and steroidogenesis, decreases inflammation, and partially restores normal ovarian function. This report is the first to present a case of a long-term amenorrheic woman with PCOS who has been trying to conceive, who resumed spontaneous ovulatory cycles, and had improvement in several aspects of her hormonal imbalance following intraovarian PRP administration. The purpose of this case report is to increase awareness regarding the possible benefits of intraovarian PRP injections for women with PCOS. There is a clear need for larger prospective studies to properly elucidate the effect of intraovarian PRP administration on both the reproductive and metabolic dysfunctions observed in women with PCOS.

## Introduction

Polycystic ovary syndrome (PCOS) is a condition affecting up to 15% of reproductive-aged women [1]. The status quo pertaining to treatment options for women with PCOS is to prescribe oral contraceptive pills for those who do not desire conception or to provide ovulation induction with or without assisted reproductive technology (ART) for those who desire pregnancy. Success with oral contraceptive pills can be somewhat limited because they can have significant side effects and are contraindicated in women with serious medical conditions. Another factor contributing to the lack of success of drugs targeting ovulation is that they do not treat the underlying etiology of ovulatory dysfunction. With respect to those women with PCOS seeking pregnancy, the use of ART is costly and often associated with complications such as multiple gestation and ovarian hyperstimulation syndrome.

Platelet-rich plasma (PRP) represents approximately 10% of the volume of whole blood, with 5 to 10 times’ higher concentration for growth factors. Platelets contain alpha granules that, once activated, release many factors that contribute to growth, cell proliferation, and angiogenesis [2]. The growth factors present in PRP play an important role in enhancing immune cell chemotaxis, macrophage activation, angiogenesis, migration and mitosis of endothelial cells, differentiation of epithelial cells, and cytokine secretion by mesenchymal and epithelial cells. It is well known that folliculogenesis is tightly regulated by a milieu of growth factors and hormones that work in paracrine and autocrine actions in the ovary [3].

The clinical use of PRP has considerably increased as an alternative therapy for several disorders including infertility [4]. Recently, PRP treatment has been used as an adjunct in ART, in particular in women who have poor ovarian reserve, premature ovarian insufficiency, and early menopause [5]. Even though women with PCOS tend to have elevated serum markers of ovarian reserve, the use of PRP in this setting has been understudied. Interestingly, a recent study that evaluated the effect of intraovarian PRP administration in a murine induced–PCOS model demonstrated improvement in folliculogenesis by diminishing PCOS-related follicular atresia and mRNA damage, decrease in PCOS-induced hormonal imbalance by decreasing serum testosterone (T), LH, FSH and androstenedione, and significant increase in estradiol (E2) and progesterone (P4) production, as well as increase in estrogen receptor (ER) expression and enhanced ovarian antioxidant potential [6].

To our knowledge, there are no studies in humans that assessed the effect of intraovarian PRP administration on ovulatory dysfunction and hormonal imbalance in women with PCOS. We present here a case where the administration of autologous intraovarian PRP demonstrated a resumption of spontaneous ovulation and improvement in hormonal imbalances in a long-term amenorrheic woman known to have PCOS.

## Case Presentation

The patient is a 35-year-old nulliparous woman who was diagnosed at aged 20 years with PCOS with clinical hyperandrogenism (facial acne and hair growth on the chin only), prolonged amenorrhea, type 2 diabetes mellitus, hypertension, and obesity (body mass index = 39 kg/m^2^). She was taking dulaglutide 1.7 mg once per week for the type 2 diabetes mellitus and losartan/hydrochlorothiazide 100/25 mg every evening with spironolactone 50 mg twice per day for hypertension.

She reported irregular periods since menarche with prolonged periods of amenorrhea that, at times, lasted for more than 1 year. She presented to our fertility center with the chief complaint of inability to conceive. She was never evaluated or treated at any previous fertility clinic before her presentation to our clinic.

## Diagnostic Assessment

The patient underwent transvaginal ultrasound examination and blood draw for measurement of blood urea nitrogen, creatinine, total T, SHBG, LH, FSH, anti-müllerian hormone (AMH), fasting glucose, dehydroepiandrosterone-sulfate, 17-hydroxyprogesterone, and high-sensitivity C-reactive protein. Her ultrasound revealed enlarged ovaries with more than 15 antral follicles per ovary. The results of her baseline blood testing are reported in Table 1. The remainder of the patient and her partner's infertility workup was within normal limits, including a prolactin level of 12.3 ng/mL (39.85 nmol/L) and TSH of 1.14 µIU/mL (1.14 µU/L).

**Table 1.** Laboratory serum values on the day of intraovarian PRP injection (baseline) and 4 weeks later

Laboratory test	Serum level at baseline	Serum level 4 weeks after PRP	Normal range
Glucose, mg/dL (nmol/L)	142 (7.88)	144 (7.99)	70-99 (3.89-5.49)
Creatinine, mg/dL (nmol/L)	1.12 (85.40)	0.95 (72.44)	0.49-1.02 (37.36-77.78)
Blood urea nitrogen, mg/dL (mmol/L)	58.82 (21)	78.43 (28)	16.81-67.23 (6-24)
High sensitivity C-reactive protein, mg/L (mg/L)	44.4 (444.00)	35.6 (356.00)	1.0-3.0 (10-30)
Total testosterone, ng/dL (nmol/L)	140.3 (4.87)	20.8 (0.72)	8.4-48.1 (0.29-1.67)
SHBG, µg/mL (nmol/L)	1.57 (14)	1.57 (14)	2.02-16.19 (18-144)
17-Hydroxyprogesterone, ng/dL (pmol/L)	218 (6047.32)	54 (1497.96)	10-125 (277.40-3467.50)
Anti-Müllerian hormone, ng/mL (mmol/L)	2.27 (7.22)	2.46 (7.82)	0.12-4.76 (0.38-15.14)

## Treatment

Before attempting any ART treatments, the couple wanted to try a method without exogenous medications because of her multiple medical problems and their fear of fertility drugs; thus, the potential use of intraovarian PRP administration was discussed, for which the patient consented in writing.

PRP was prepared as we previously described [7]. Approximately 40 mL of blood was collected from the patient by peripheral venipuncture. After centrifugation (1500*g* for 5 minutes), the upper layer containing platelet-poor plasma was aspirated and discarded, after which the PRP layer was aspirated and placed in a separate tube for a second round of centrifugation. The process was repeated a second time. A total of 4 mL of PRP was collected from the tubes, and no activators were used. Under IV sedation and transvaginal ultrasound guidance, intraovarian injection of approximately 2 mL of PRP per ovary was performed. The injection was performed in multifocal spots and the diffusion of the PRP in the subcortical layers was achieved by applying 5 to 7 punctures per ovary transvaginally using a 22-gauge needle and guide. The patient tolerated the procedure well.

## Outcome and Follow-up

Ten days following PRP administration, a repeat transvaginal ultrasound showed a dominant follicle measuring 14.5 mm on her right ovary with mildly elevated serum E2 level (105.2 pg/mL or 23.14 pmol/L) compared with baseline (80.88 pg/mL or 17.79 pmol/L). Follow-up monitoring showed growth of that follicle to 17.5 mm 3 days later, with an increase in serum E2 level to 208.2 pg/mL (45.80 pmol/L). The patient was instructed to have timed intercourse the 2 following days. The patient returned for blood test and ultrasound 4 days later that confirmed ovulation by an elevated serum P4 level (3.05 ng/mL or 9.99 nmol/L) and the presence of a corpus luteum on her ovary. The patient returned 2 weeks following the presumed ovulation day, having her menstrual cycle. On that day, all her blood testing was repeated, and her serum pregnancy test was confirmed negative. As seen in Table 1, there were significant changes to her laboratory parameters, particularly a drop in her serum T level.

Following resumption of a normal menstrual period, the patient desired to pursue another monitored cycle, with scheduled intrauterine insemination (IUI) instead of timed intercourse. Her baseline serum hormone levels on day 1 of her cycle revealed an E2 level of 37.3 pg/mL (8.21 pmol/L), P4 of 0.47 ng/mL (1.52 nmol/L), FSH of 7.1 mIU/mL (7.1 IU/L), and LH of 2.7 mIU/mL (2.7 IU/L) with more than 20 antral follicles noted bilaterally on transvaginal ultrasound and a thin endometrial lining of 3.7 mm. On day 8 of that cycle, her E2 level was noted to have increased to 65.8 pg/mL (14.48 pmol/L) but no dominant follicle was observed on ultrasound. On cycle day 15, a dominant follicle measuring 11.5 mm on the left ovary had emerged, with a corresponding E2 of 87.1 pg/mL (19.16 pmol/L). On cycle day 19, the patient had a dominant mature follicle in the left ovary measuring 18 mm and an endometrial lining thickness of 7.8 mm with trilaminar pattern and a corresponding E2 of 154 pg/mL (33.88 pmol/L) and LH of 7.4 mIU/mL (7.4 mU/L). On cycle day 20, the patient received ovulation trigger injection of 40 units of leuprolide acetate followed by an IUI the next day. Of note, the sperm parameters on the day of the IUI were normal (105 million motile sperm). The patient did not have in-office follow-up following the procedure because she had a family engagement outside of the country; however, she reported to us by email that she experienced a menstrual period 2 weeks following the IUI. The patient's diabetes and hypertension were very well controlled before and after the procedure because she was compliant with taking her medications. The patient was lost to follow up after then.

## Discussion

This report describes the effect of intraovarian PRP administration in an amenorrheic women with PCOS showing that PRP induced follicular development with an ovulatory event. Additionally, there was a significant drop in the abnormally elevated serum T level. It was interesting that the patient developed a dominant follicle in the right ovary in 1 cycle and a dominant follicle in the left ovary in the second cycle. This complex monofollicular development was most likely the result of the physiologic follicular growth and regression rates under spontaneous conditions (ie, without ovulation induction). Even though women with PCOS have elevated AMH, those who have diabetes on medications, such as our patient here, tend to have lower than usual AMH levels (Table 1).

There has only been 1 study published to date that investigated the effect of PRP in PCOS-induced female Sprague-Dawley rats in which PRP was administered after abdominal incision into the meso-ovarian space [6]. The aim of that study was to assess the possible ameliorative effect of PRP on hyperandrogenic PCOS-induced (by dehydroepiandrosterone DHEA) derangements in ovarian tissue. The animals were then euthanized, and the entire ovary was sectioned and processed for immunohistochemical staining and analysis of mRNA expression. Compared with nontreated PCOS-induced ovaries, the results demonstrated improvements in ovarian parameters such as diminished PCOS-induced follicular atresia and mRNA damage as shown by a higher number of intact preantral and antral follicles per ovary, increased total antioxidant capacity, ERα/β expression, glutathione peroxidase, superoxide dismutase, and decreased c-Myc expression and malondialdehyde expression. The PRP also improved PCOS-induced hormonal imbalance by increasing circulating E2 and P4 as well as decreasing LH, FSH, T, and androstenedione serum levels. These data suggest that PRP can induce antiandrogenic and antioxidant effects in ovarian tissue.

A potential mechanism for the improvement of follicular development following intraovarian PRP administration could be explained by the local action of growth factors, immune modulators, and other cytokines on folliculogenesis. Prior studies have demonstrated that in humans, there is a different gene expression profile of the granulosa cells of women with PCOS compared with women without PCOS. Expression of ERα and ERβ are important elements in follicular growth/atresia. Estrogen binding to its receptor (ERα and ERβ) upregulates local growth factor secretion, which further promotes follicular maturation (IGF and epidermal growth factor). Failed ERβ expression has been shown to lead to chronic anovulation. It is plausible that PRP exerts its effect on the ovary by upregulating the expression of these genes within granulosa cells of small antral follicles, therefore potentializing their effect and preventing the follicles from undergoing atresia [6].

PRP contains several growth factors, chemokines and cytokines, immune mediators, integral membrane proteins, adhesive proteins, and clotting factors and inhibitors. Although many studies have demonstrated that intraovarian PRP administration enhances ovarian function, the specific role of particular PRP mediators and exact mechanism of actions remain unclear. The growth factors found in PRP include TGF-β, vascular endothelial growth factor, IGFs, platelet-derived growth factor (PDGF), epidermal growth factor, basic fibroblast growth factor, fibroblast growth factor 2, and hepatocyte growth factor, each of which has been shown to play a role in the potential modulation of ovarian function [2, 8]. Both in vitro and in vivo studies have demonstrated positive effects of IGFs on oocyte maturation; this is mainly because of an effect on LH signaling for oocyte maturation, differentiation of granulosa cells, and increasing responsiveness of the ovary to FSH action [9]. Additionally, the potential antioxidant effect of PRP could have a boosting effect on follicular growth, keeping in mind that antioxidant chemicals are used to manage or reduce the PCOS-induced pathogenesis. Consistently, various studies have shown that administrating antioxidant agents can potentially improve insulin sensitivity and enhance the ovarian antioxidant potential in women with PCOS.

Insulin resistance in women with PCOS has been implicated in a higher oxidative stress, and IGF has been shown to promote the selection of dominant follicle and inhibition of androgen steroidogenesis in the theca cells of subordinate follicles, which could explain the drop in T levels observed in our patient. Androgen levels may also be influenced by improved antioxidant capacity by means of decreased total antioxidant capacity and decreased lipid peroxidation ratio after injection of PRP, which was demonstrated in an induced PCOS murine model [6]. The TGF-β superfamily of growth factors has a key role in promoting folliculogenesis. Bone morphogenic proteins, which are part of the TGF-β superfamily and found in PRP, have a role in the ovary by attenuating the production of androgen in the theca cells of smaller-to-medium sized, nondominant follicles, which are aberrantly prevalent in women with PCOS. GDF-9 and bone morphogenic protein-15, which are present in PRP, also act to sensitize the dominant follicle to FSH. PDGFs also have been demonstrated to affect follicular development by exhibiting an autocrine and paracrine effect on granulosa and theca cells and their mediation by PDGF receptors [8, 10].

It is important to consider other factors as potential limitations that may have led to the spontaneous ovulation in our patient. Even though unlikely, it may have been coincidental that she resumed ovulation on her own. By evaluating her second cycle, we noted that it took a few days longer to develop a mature follicle, which may demonstrate that there is a possible lasting, yet diminishing effect of PRP injection over time. Another mechanism to consider is the possible mechanical effect of injecting the needle into the ovary, mimicking a miniscule needle drilling effect; laparoscopic ovarian drilling, also known as ovarian diathermy, which has been used for resuming spontaneous cycles in women with PCOS, by implementing a larger bore needle with monopolar current /or laser for its treatment. We have used in our case a very thin 22-gauge needle, which is unlikely to have caused the same effect as ovarian drilling, which uses much bigger gauge.

The purpose of this case report is to increase awareness to the potential effects of intraovarian PRP administration on folliculogenesis in PCOS setting. The experience in this single patient should trigger larger prospective studies with a longer follow-up to properly elucidate the effect of intraovarian PRP administration on both the reproductive and metabolic dysfunctions observed in women with PCOS.

## Learning Points

- PRP is hypothesized to affect folliculogenesis in the ovary by directly applying growth factors and cytokines into the ovarian tissue.
- PRP has been used in reproductive medicine via intrauterine infusion in women with recurrent implantation failure and via intraovarian administration in women with low ovarian reserve.
- This report suggests that PRP may have potential beneficial effects for women with ovulatory disorders and hormonal imbalances such as PCOS.

## Contributors

Z.M., S.S., M.M., and R.W. were involved in the diagnosis and management of this patient, literature search, manuscript writing, and editing and manuscript submission. Z.M. performed the procedure on the patient. All authors reviewed and approved the final draft.

## Data Availability

All data generated and analyzed during this report are included in this published article.

## Abbreviations

AMH: anti-müllerian hormone
ART: assisted reproductive technology
E2: estradiol
ER: estrogen receptor
IUI: intrauterine insemination
P4: progesterone
PCOS: polycystic ovary syndrome
PDGF: platelet-derived growth factor
PRP: platelet-rich plasma
T: testosterone
